# 1-Aminoalkylphosphonium Derivatives: Smart Synthetic Equivalents of *N*-Acyliminium-Type Cations, and Maybe Something More: A Review †

**DOI:** 10.3390/molecules27051562

**Published:** 2022-02-26

**Authors:** Jakub Adamek, Mirosława Grymel, Anna Kuźnik, Agnieszka Październiok-Holewa

**Affiliations:** 1 Department of Organic Chemistry, Bioorganic Chemistry and Biotechnology, Silesian University of Technology, B. Krzywoustego 4, 44-100 Gliwice, Poland; miroslawa.grymel@polsl.pl (M.G.); anna.kuznik@polsl.pl (A.K.); agnieszka.pazdzierniok-holewa@polsl.pl (A.P.-H.); 2 Biotechnology Center, Silesian University of Technology, B. Krzywoustego 8, 44-100 Gliwice, Poland; 3 Department of Chemical Organic Technology and Petrochemistry, Faculty of Chemistry, Silesian University of Technology, B. Krzywoustego 4, 44-100 Gliwice, Poland

**Keywords:** phosphonium salts, *N*-acyliminium cations, α-amidoalkylation, α-amidoalkylating agents, ylides, Wittig reaction

## Abstract

*N*-acyliminium-type cations are examples of highly reactive intermediates that are willingly used in organic synthesis in intra- or intermolecular α-amidoalkylation reactions. They are usually generated in situ from their corresponding precursors in the presence of acidic catalysts (Brønsted or Lewis acids). In this context, 1-aminoalkyltriarylphosphonium derivatives deserve particular attention. The positively charged phosphonium moiety located in the immediate vicinity of the *N*-acyl group significantly facilitates C_α_-P^+^ bond breaking, even without the use of catalyst. Moreover, minor structural modifications of 1-aminoalkyltriarylphosphonium derivatives make it possible to modulate their reactivity in a simple way. Therefore, these types of compounds can be considered as smart synthetic equivalents of *N*-acyliminium-type cations. This review intends to familiarize a wide audience with the unique properties of 1-aminoalkyltriarylphosphonium derivatives and encourage their wider use in organic synthesis. Hence, the most important methods for the preparation of 1-aminoalkyltriarylphosphonium salts, as well as the area of their potential synthetic utilization, are demonstrated. In particular, the structure–reactivity correlations for the phosphonium salts are discussed. It was shown that 1-aminoalkyltriarylphosphonium salts are not only an interesting alternative to other α-amidoalkylating agents but also can be used in such important transformations as the Wittig reaction or heterocyclizations. Finally, the prospects and limitations of their further applications in synthesis and medicinal chemistry were considered.

## 1. Introduction

**Scheme 1:**
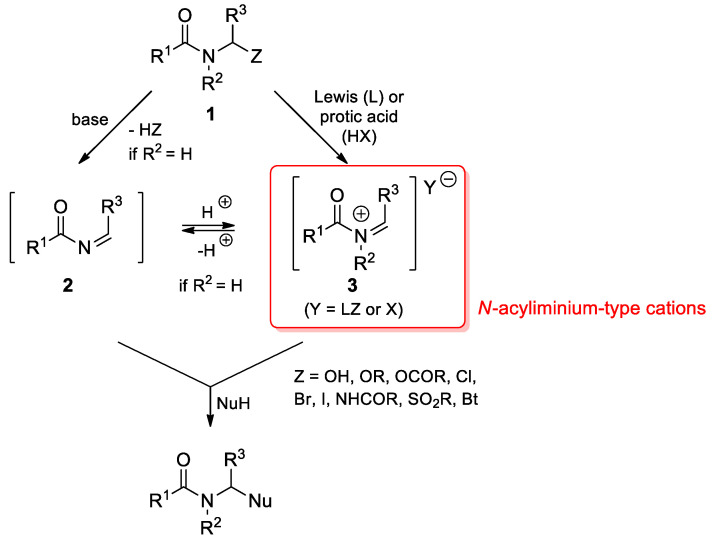
The α-amidoalkylation reaction.

α-Amidoalkylation reactions play an increasingly important role in organic synthesis as convenient and effective methods for the formation of C-C and C-heteroatom bonds, particularly of the intramolecular type, allowing the synthesis of carbo- or heterocyclic systems. In most cases, *N*-acylimine **2** or *N*-acyliminium cations **3** are the correct α-amidoalkylating agents and they are generated from precursors with the relevant structure **1** (Scheme 1) [1,2,3,4,5,6,7,8,9,10,11,12,13,14,15,16,17,18,19,20,21,22,23].

Many examples of α-amidoalkylating agent precursors and their applications in α-amidoalkylations have been reported in the literature. A brief summary is given in Table 1. Compared to the precursors described therein, 1-aminoalkylphosphonium derivatives are relatively unknown compounds. However, they have unique structural features which promote the generation of *N*-acyliminium-type cations. One of the most important is the presence of a positively charged phosphonium moiety (which easily departs as triarylphosphine PAr_3_) in the immediate vicinity of the acyl group.

**Figure 1:**
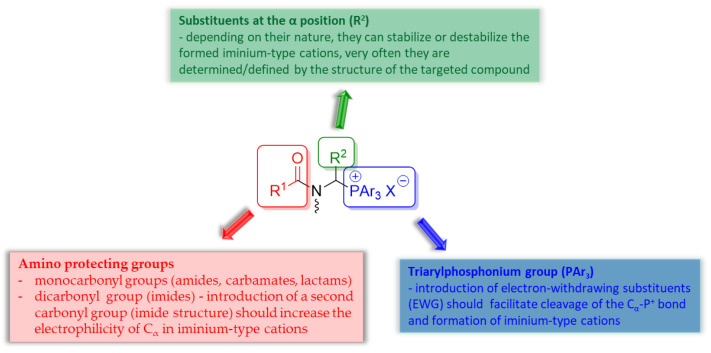
Areas of potential structural modifications within phosphonium precursors of α-amidoalkylating agents.

Moreover, the reactivity of 1-aminoalkylphosphonium derivatives can be modulated by simple structural modifications, e.g., by changing the amino protecting group or by the introduction of electron-withdrawing substituents to the phosphonium moiety (replacing Ph_3_P by (3-C_6_H_4_Cl)_3_P or (4-C_6_H_4_CF_3_)_3_P; see Figure 1). Depending on the structure of the phosphonium salt used, the α-amidoalkylations may require a basic or acidic catalyst. However, the introduction of the abovementioned activating structural modifications allows one, in many cases, to conduct the reactions under milder and even catalyst-free conditions. Furthermore, such modifications not only affect the reactivity but also the course of the reaction (for example, to reduce side reactions), or even make it possible to change the type of reaction taking place (the α-amidoalkylation reaction vs the Wittig reaction).

**Scheme 2:**
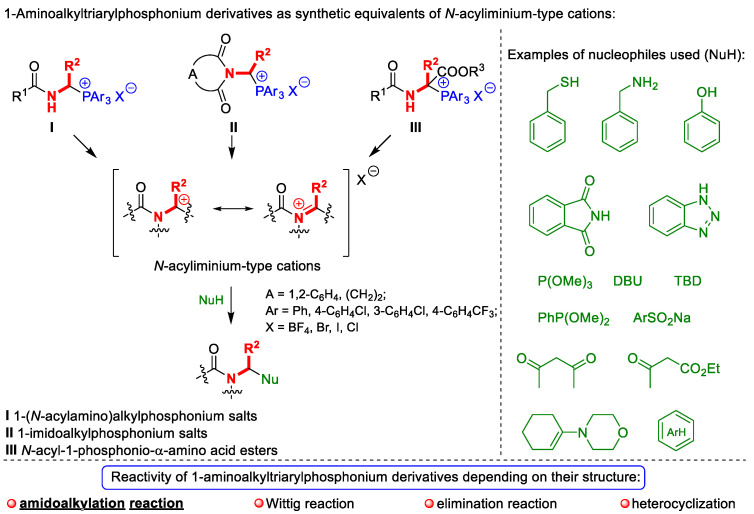
Classification and reactivity of 1-aminoalkylphosphonium derivatives.

The main purpose of this review paper is to organize and disseminate current knowledge about 1-aminoalkylphosphonium derivatives. To help understand the presented issues, three classes of these P-compounds have been distinguished. Three separate chapters are dedicated to them, where general properties, the most important methods for preparation as well as synthetic applications are described. Particularly, the correlation between the structure and the reactivity of phosphonium derivatives I-III is discussed. Scheme 2 provides a classification and a brief summary of the chemistry of 1-aminoalkylphosphonium derivatives.

## 2. 1-Aminoalkyltriarylphosphonium Derivatives

### 2.1. 1-(N-acylamino)alkylphosphonium Salts

**Figure 2:**
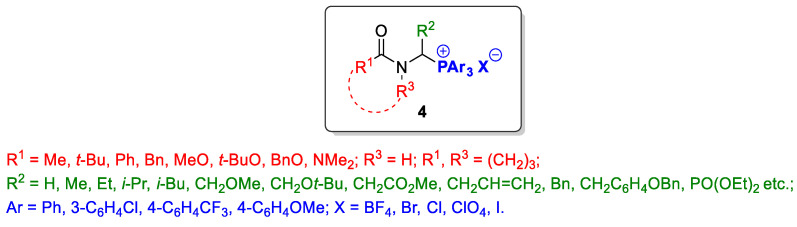
General structure of 1-(*N*-acylamino)alkylphosphonium salts **4**.

Compounds with general formula **4** (Figure 2) are often called 1-(*N*-acylamino)alkylphosphonium salts, because a lot of the described models are amide derivatives (e.g., R^1^ = H, Me, Et, *t*-Bu, Ph, Bn, etc.; R^3^ = H). It is not an exact name because this group also includes lactams (e.g., R^1^, R^3^ = (CH_2_)_3_), carbamates (R^1^ = *t*-BuO, BnO; R^3^ = H) or urea derivatives (e.g., R^1^ = NMe_2_, R^3^ =H). In the α-position, there may be hydrogen (R^2^ = H), alkyl (R^2^ = Me, Et, *i*-Bu, etc.), aryl (R^2^ = Ph, 2-thienyl, 1-naphtyl, etc.) or more complex substituents (e.g., CH_2_CO_2_-*t*-Bu, CH_2_C_6_H_4_OBn, PO(OEt)_2_ etc.). The positively charged triarylphosphonium group PAr_3_ (Ar = Ph, 3-C_6_H_4_Cl, 4-C_6_H_4_CF_3_) is also directly bonded to C_α_.

1-(*N*-acylamino)alkyltriphenylphosphonium salts **4** (Ar = Ph) are crystalline, stable at room temperature compounds that can be stored under laboratory conditions for a long time. They are well soluble in DCM and MeCN, but insoluble in diethyl ether. The most effective method of their purification is crystallization from DCM/Et_2_O or MeCN/Et_2_O systems. 1-(*N*-acylamino)alkyltriarylphosphonium salts **4** which are derivatives of triarylphosphines with electron-withdrawing substituents (Ar = 3-C_6_H_4_Cl or 4-C_6_H_4_CF_3_) are less stable. They are usually synthesized just before the reaction and used without purifiaction. The type of phosphonium group used has a huge impact on the reactivity of the whole molecule, which will be discussed later in this review.

#### 2.1.1. Preparation

**Scheme 3:**
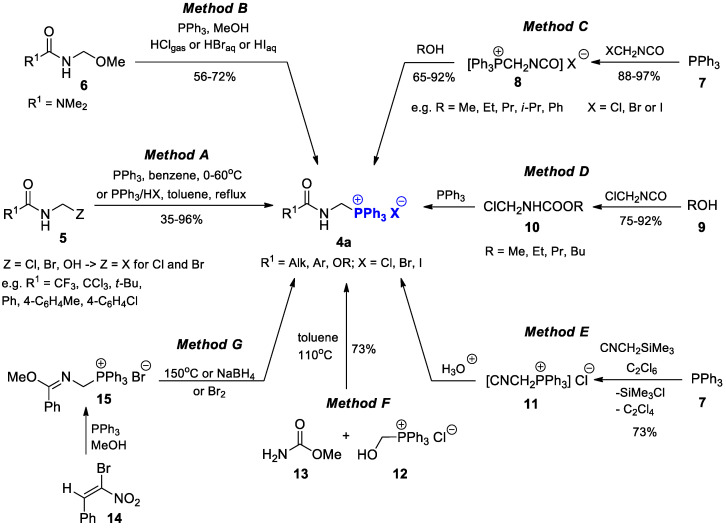
Methods for the synthesis of 1-(*N*-acylamino)methyltriphenylphosphonium salts **4a**.

In the last century, most of the methods for the synthesis of 1-(*N*-acylamino)alkyltriarylphosphonium salts **4** concerned 1-(*N*-acylamino)methyltriphenylphosphonium salts (**4a**, R^2^ = H, Scheme 3). Between 1972 and 1991, Drach, Brovarets and co-workers [24,25,26,27] showed that 1-(*N*-acylamino)methylphosphonium chlorides (**4a**, X = Cl) can be obtained, in a simple reactions, by alkylation of triphenylphosphine (but also tributylphosphine PBu_3_ or hexaethylphosphorus triamide P(NEt_2_)_3_) with *N*-(chloromethyl)amides (**5**, Z = Cl) (Scheme 3, Method A). They also used *N*-(hydroxymethyl)amides (**5**, Z = OH) as alkylating agents, that were *N*-(chloromethyl)amides precursors (Scheme 3, Method A) [27]. In 1974, Devlin and Walker reported similar reactions, which were carried out at room temperature, using AcOEt as a solvent. They obtained 1-(*N*-benzoylamino)methyltriphenylphosphonium bromide or chloride (**4a**, X = Br or Cl) from *N*-(bromomethyl)benzamide or *N*-(chloromethyl)benzamide, respectively, in 54% and 69% yield (Scheme 3, Method A) [28]. Triphenylphosphine was also alkylated with *N*-(methoxymethyl)urea derivative **6** (Scheme 3, Method B). Reactions were carried out in methanol by bubbling HCl gas through the substrate solution or by treating it with aqueous HBr or HI [29]. 1-(*N*-alkoxycarbonyl)methyltriphenylphosphonium chlorides or bromides (**4a**, R^1^ = OR, X = Cl or Br) were obtained by Kozhushko et al. in the reaction of triphenylphosphine with chloromethylisocyanate or bromomethylisocyanate and further hydrolysis of the isocyanate group (Scheme 3, Method C) [30,31]. In analogous reactions, the corresponding triphenylphosphonium iodides (**4a**, R^1^ = OR, X = I) were also obtained by adding methyl iodide in the first step of the synthesis [32]. The same authors also described reactions in which phosphonium salts **4a** (R^1^ = OR, X = Cl) were obtained by alkylation of triphenylphosphine with *N*-(chloromethyl)carbamates **10**, that were previously generated from alcohol and methyl isocyanide (Scheme 3, Method D) [33]. In turn, Zinner and Fehlhammer described the two-stage method for the synthesis of 1-(*N*-formylamino)methyltriphenylphosphonium chloride **4a** (R^1^ = H, X = Cl). Initially, they conducted the alkylation of triphenylphosphine using trimethylsilyl isocyanide in the presence of hexachloroethane in THF. The acidic hydrolysis of indirectly formed isocyanomethyltriphenylphosphonium chloride **11** finally yielded the expected phosphonium salt **4a** (Scheme 3, Method E) [34]. However, the authors did not report the yield of the hydrolysis step.

Only a few of the described methods for synthesizing 1-(*N*-acylamino)methyltriphenylphosphonium salts **4a** were based on other approaches than the alkylation of triphenylphosphine by *N*-(halomethyl)amides, their precursors or related compounds. One of these methods involved the alkylation of methyl carbamate with hydroxymethyltriphenylphosphonium chloride **12**, which resulted in the production of 1-(*N*-methoxycarbonyl)aminomethyltriphenylphosphonium chloride **4a** (R^1^ = OMe, X = Cl) in 73% yield (Scheme 3, Method F) [35]. Devlin and Walker demonstrated that the treatment of 2-bromo-2-nitrostyrene **14** with triphenylphosphine in methanol gave the phosphonium salt **15** in 47% yield. The vacuum pyrolysis of salt **15** at 150 °C, reduction with NaHBF_4_ in methanol or refluxing in chloroform with addition of bromine led to a mixture containing 1-(*N*-benzoylamino)methyltriphenylphosphonium bromide **4a** (R^1^ = Ph, X = Br) as the main product (Scheme 3, Method G) [28,36].

**Scheme 4:**
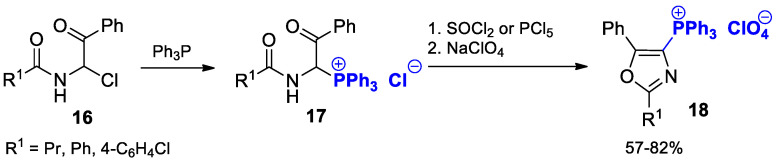
Synthesis of 1-(*N*-acylamino)benzoylmethyltriphenylphosphonium chlorides **17**.

There are few data available in the literature on the synthesis of 1-substituted phosphonium salts **4**. In 1975, Drach et al. demonstrated that the reaction of triphenylphosphine with *N*-(1-benzoyl-1-chloromethyl)amides **16** led to triphenylphosphonium salts **17** with a benzoyl group at the 1-position. However, salts **17** turned out to be hygroscopic and unstable. Thus, the authors decided to transform them into more stable oxazolones **18** (Scheme 4) [37].

**Scheme 5:**
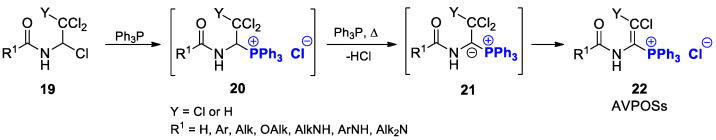
Synthesis of 1-(*N*-acylamino)vinylphosphonium salts (AVPOSs) **22**.

Next, Drach et al. described the route for the synthesis of various 1-(*N*-acylamino)-substituted vinylphosphonium salts **22**, which was based on the condensation of triphenylphosphine with *N*-polychloroalkylamides **19** [38,39]. As reported by the authors, in the first step, the salts **20** were probably formed, which further split off hydrogen chloride, resulting in the formation of the corresponding vinylphosphonium salts **22**, typically in yields above 90% (Scheme 5). 1-(*N*-acylamino)vinylphosphonium salts (AVPOSs) **22** are unique reagents for various types of heterocyclization, which was comprehensively discussed by Drach, Brovarets, and co-workers in 2002 [39].

At about the same time, Mazurkiewicz et al. started more extensive research on the synthesis of structurally diverse 1-(*N*-acylamino)alkyltriarylphosphonium salts **4**. Wherein, the common feature of these methods was the raw materials, which was *N*-protected α-amino acids. The use of α-amino acids or their derivatives as substrates was greatly advantageous, due to almost unlimited availability and structural diversity of such compounds.

**Scheme 6:**
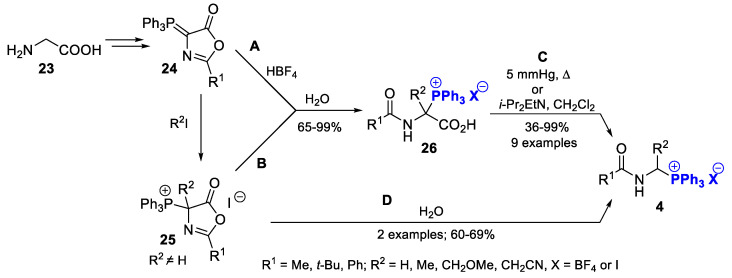
Synthesis of 1-(*N*-acylamino)alkylphosphonium salts **4** from oxazolones.

The first approach was based on using 4-triphenylphosphoranylidene-5(4*H*)-oxazolones **24** or 4-alkyl-4-triphenylphosphonio-5(4*H*)-oxazolones **25**, obtained from glycine (Scheme 6) [40]. Phosphoranylidene-5(4*H*)-oxazolones **24**, were hydrolyzed at room temperature in the presence of HBF_4_ to *N*-acyl-α-triphenylphosphonioglycines **26** (R^2^ = H, Scheme 6/A). Similarly, phosphonium iodides **25** were exposed to water in the mixture of THF/DCM, but without any acidic catalyst. Under these conditions, compounds **25** were transformed, in a few days, into *N*-acyl-1-triphenylphosphonio-α-amino acids **26** (R^2^ = Me, Scheme 6/B). In the next stage, 1-triphenylphosphonio-α-amino acids **26** were heated at 105–115 °C under reduced pressure (5 mmHg) or treated with diisopropylethylamine in DCM at 20 °C, which resulted in their decarboxylation to corresponding 1-(*N*-acylamino)alkyltriphenylphosphonium salts **4**, usually in good yields (Scheme 6/C). The authors also showed, that in the case of hydrolysis of 4-alkyl-4-triphenylphosphonio-5(4*H*)-oxazolones **25** with a bulky substituent in the 4-position, the reaction proceeded with simultaneous decarboxylation and gave the expected 1-(*N*-acylamino)alkyltriphenylphosphonium salts **4** in one reaction step (Scheme 6/D) [41,42].

**Scheme 7:**
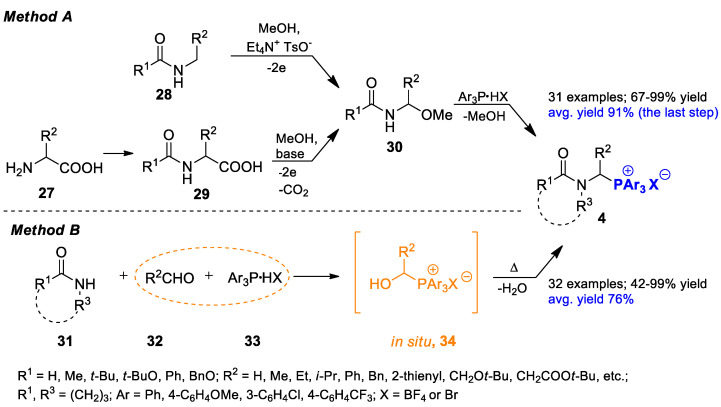
Modern strategy in the synthesis of 1-(*N*-acylamino)alkylphosphonium salts **4**; Method A–Synthesis based on the electrochemical alkoxylation; Method B–Non-electrochemical synthesis based on the one-pot, three components coupling.

However, the two most important and general methods for the synthesis of 1-(*N*-acylamino)alkylphosphonium salts **4** were developed by Mazurkiewicz and Adamek in the last 10 years (Scheme 7) [43,44].

The first, three-stage method begins with the appropriate protection of α-amino acid functional groups (the NH_2_ group and other groups susceptible to electrochemical oxidation). Next, electrochemical decarboxylative α-methoxylation (or more generally, alkoxylation) takes place. As the authors noted, the electrochemical oxidations could be carried out in methanol with the addition of sodium methoxide as a base or in the presence of a solid-supported base (SiO_2_-Pip); wherein the latter process (based on a solid-supported base) proceeded in excellent yields and had a less complicated work-up. Recently, a simpler and even more efficient, standardized method for preparation of *N*,*O*-acetals **30** using the commercially available ElectraSyn 2.0 setup (graphite electrodes, Et_3_N as a base, room temp.) was described [45].

The last step is the substitution of the methoxy group in the reaction of *N*,*O*-acetals **30** with various types of phosphonium salts (Ar_3_P·HX, Scheme 7; Method A). The proposed method allows high yields (up to 99%) to be obtained not only for the simplest 1-(*N*-acylamino)alkylphosphonium salts **4** (e.g., R^2^ = H), but also for much more complex structure, including derivatives of phosphine with various substituents (Ar = Ph, 3-C_6_H_4_Cl, 4-C_6_H_4_CF_3_) [43,46]. Moreover, the raw material base can be expanded, since *N*-methoxyalkyl derivatives can be obtained by electrochemical oxidation of amides, carbamates or lactams. However, this is a less efficient process and an aqueous work-up of the reaction mixture is necessary [47].

In 2021, a procedure for the prepartion of *N*-protected aminoalkylphosphonium salts (including 1-(*N*-acylamino)alkylphosphonium ones) in one reaction step from aldehydes and either amides, carbamates, lactams, or urea in the presence of phosphonium salts **33** -Ar_3_P·HX (Scheme 7; Method B) was described [44]. Using a one-pot methodology, the simple work-up of the reaction mixture (no chromatography) makes 1-(*N*-acylamino)alkylphosphonium salts obtainable in high yields under relatively mild conditions (even at room temperature, but usually at 50 °C for 1 h). So far, it is the only general method of obtaining *N*-protected aminoalkylphosphonium salts without the use of electrochemical techniques [44]. Mechanistic studies showed that in the first step of the transformation, aldehydes and phosphonium salts (Ar_3_P·HX) form 1-hydroxyalkylphosphonium salts **34**, which then react with amide-type substrates **31** to give the desired 1-(*N*-acylamino)alkylphosphonium salts **4** in good to excellent yields [44].

**Scheme 8:**
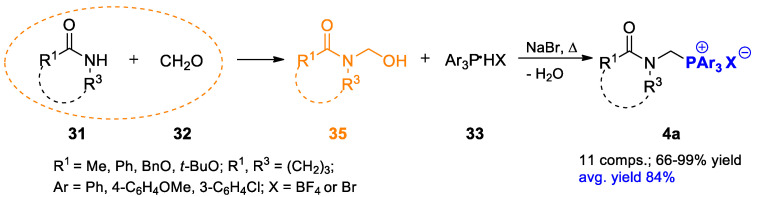
Step-by-step procedure for the synthesis of *N*-protected aminomethylphosphonium salts **4a**.

Next, it was shown that by conducting the reaction step-by-step and changing the order of the reacting compounds, 1-(*N*-acylamino)alkylphosphonium salts **4** could also be obtained. However, the procedure is effective only for formaldehyde (or paraformaldehyde). Hydroxymethylamides **35**, already mentioned in the introduction (see also Table 1), are generated during such a transformation (Scheme 8). This method works well for the synthesis of *N*-protected aminomethyltriarylphosphonium salts **4a**, but requires a catalyst (NaBr) and elevated temperatures (70–135 °C) [48].

The presented methods (Scheme 7 and Scheme 8) are based on a wide and diverse base of raw materials (α-amino acids, amide-type compounds, aldehydes), and provide easy access to structurally diverse 1-(*N*-acylamino)alkylphosphonium salts **4** also in the synthesis on a larger gram-scale [44,48].

#### 2.1.2. Synthetic Utilization

**Figure 3:**
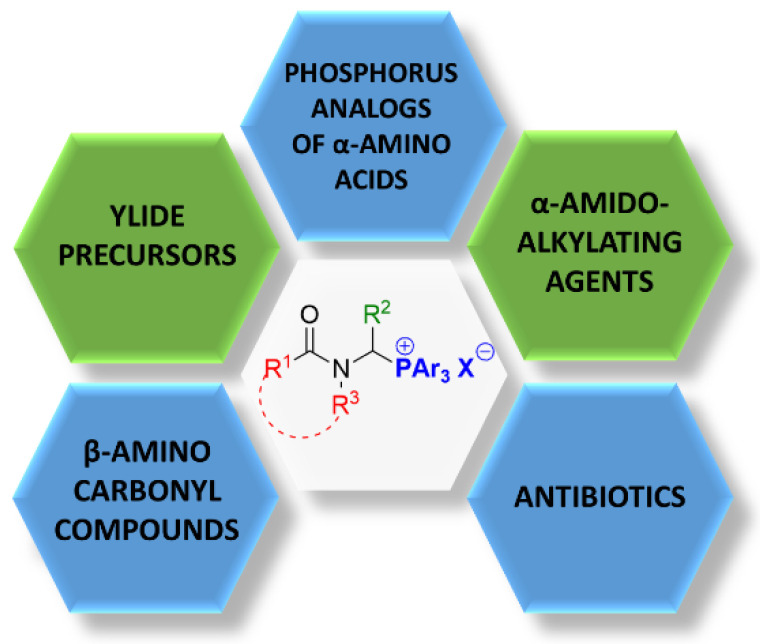
Applications of 1-(*N*-acylamino)alkylphosphonium salts **4**.

**Scheme 9:**
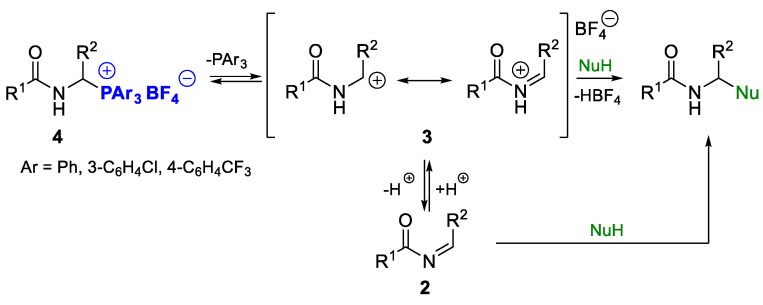
1-(*N*-acylamino)alkyltriarylphosphonium salts **4** as precursors of *N*-acylimines **2** and *N*-acyliminium-type cations **3**.

Synthetic applications of 1-(*N*-acylamino)alkylphosphonium salts **4** are summarized in Figure 3. The high reactivity of such compounds is mainly related to the possibility of easy cleaving of the C_α_-P^+^ bond (Scheme 9).

**Scheme 10:**
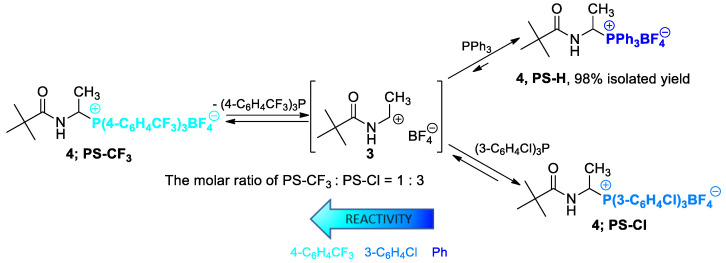
Generation of *N*-acyliminium-type cations from 1-(*N*-acylamino)alkyltriarylphosphonium salts **4**.

The strength of the C_α_-P^+^ bond can be further reduced by introducing electron-withdrawing substituents to the phosphonium moiety (Scheme 10, Ar = 3-C_6_H_4_Cl and 4-C_6_H_4_CF_3_). The equilibrium in such systems was examined and described in 2018 [46]. As can be seen, it is shifted toward more stable and less reactive 1-(*N*-acylamino)alkylphosphonium salts (reactivity: PS-CF_3_ > PS-Cl > PS-H; stability: PS-CF_3_ < PS-Cl < PS-H).

The ease of formation of iminium-type cations **3** from phosphonium salts **4** was essential in the α-amidoalkylation reactions of various types of nucleophiles (*C*-nucleophiles and heteronucleophiles). In many cases, the generation of such reactive intermediates can proceed without the use of any catalysts, which is an amazing advantage compared to other α-amidoalkylating agents described in the literature (e.g., *N*-(1-methoxyalkyl)amides, α-amido sulfones, or *N*-(benzotriazolylalkyl)amides) [12,20].

One of the most widely described α-amidoalkylation reactions involving 1-(*N*-acylamino)alkylphosphonium salts is the reaction with *P*-nucleophiles: phosphites, phosphonites, or phosphinites. The products of these transformations are called phosphorus analogs of α-amino acids **37** (more precisely: 1-aminoalkanephosphonic acid derivatives, 1-aminoalkanephosphinic acid derivatives, or 1-aminoalkylphosphine oxide derivatives), and they are extremely interesting in terms of their biological activity [49].

**Scheme 11:**
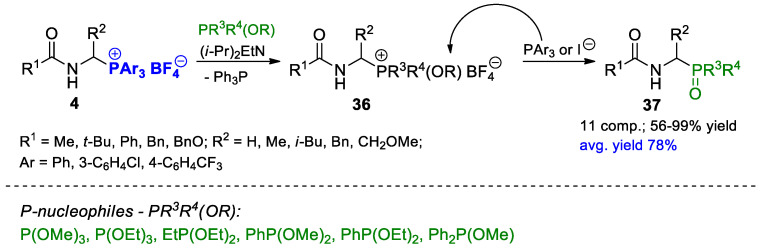
Michaelis–Arbuzov-type reaction of 1-(*N*-acylamino)alkylphosphonium salts **4** with *P*-nucleophiles.

Initially, the Michaelis–Arbuzov-type reaction with a double catalytic system was used for the synthesis of such compounds. A base (e.g., the Hünig’s base-(*i*-Pr)_2_EtN) facilitates the cleavage of the C_α_-P^+^ bond and the formation of corresponding *N*-acylimine. In turn, the iodide anion (introduced as methyltriphenylphosphonium iodide) enables dealkylation of the intermediate alkoxyphosphonium salt **36** (Scheme 11) [50,51,52]. Further studies showed that the reaction could be carried out also under a catalytic-free conditions [46,52]. It was also possible, for the first time, to isolate and characterize one of the intermediates **36** (R^1^ = *t*-Bu; R^2^ = Me; R^3^, R^4^ = OR = OEt, Scheme 11), thus proving the reaction mechanism [46].

**Scheme 12:**
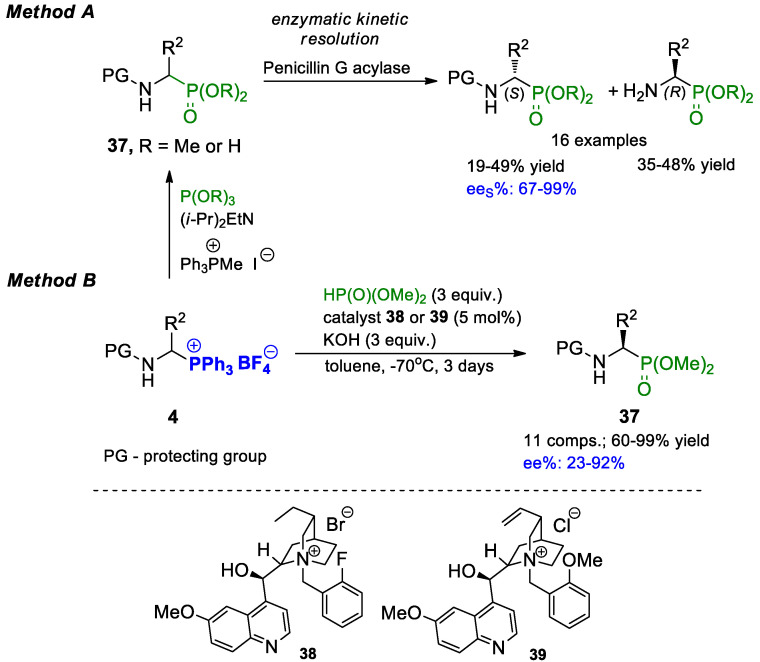
Methods for the obtaining of enantiomerically enriched phosphorus analogs of α-amino acids **37** via 1-(*N*-acylamino)alkyltriphenylphosphonium salts **4** based on enzymatic kinetic resolution (Method A) or organocatalytic α-amidoalkylation of *P*-nucleophiles in PTC systems (Method B).

Unfortunately, the major disadvantage of these reactions is the complete racemization of the products. However, two solutions were proposed to overcome this drawback. The first was enzymatic kinetic resolution of products using Penicillin G acylase from *Escherichia coli* (Scheme 12, Method A) [53,54]. The second was changing the synthetic approach and to conduct organocatalytic α-amidoalkylation of *P*-nucleophiles (e.g., dimethyl phosphite; Michaelis–Becker-type reaction) by 1-(*N*-acylamino)alkyltriphenylphosphonium salts in PTC systems using Cinchona alkaloid derivatives **38** and **39** as catalysts (Scheme 12, Method B) [55].

**Scheme 13:**
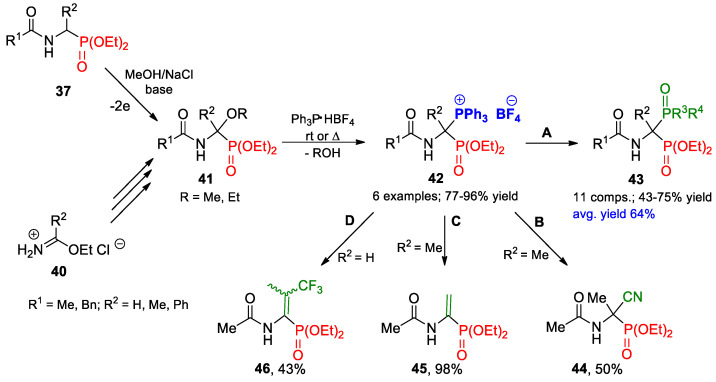
Synthesis and applications of 1-(*N*-acylamino)-1-triphenylphosphoniumalkylphosphonates **42**. Reagents and conditions: (**A**) R^3^R^4^POR (e.g., P(OEt)_3_, MeP(OEt)_2_, Ph_2_P(OMe), etc.), (*i*-Pr)_2_EtN, Ph_3_P^+^Me I^−^, 20–60 °C, 0.3–6 h; (**B**) KCN, 18–crown–6, 20 °C, 24 h; (**C**) (*i*-Pr)_2_EtN, 20 °C, 5 h; (**D**) MeC(O)CF_3_, K_2_CO_3_, 18-crown-6, 50 °C, 4 h.

Further research on phosphorus analogs of α-amino acids **37** revealed the possibility of transforming them into bisphosphoric acid esters **43**, which also exhibit important biological activity (Scheme 13) [56,57].

Electrochemical alkoxylation of compounds **37** followed by substitution of the alkoxy group leads to 1-(*N*-acylamino)-1-triphenylphosphoniumalkylphosphonates **42**. They can be also synthesized in a multi-stage procedure from imidate hydrochlorides **40** (Scheme 13).

As shown, the high reactivity of the phosphonium salts **42** can be used not only in the α-amidoalkylation reactions of phosphorus or carbon nucleophiles (Scheme 13, route A and B) but also in the elimination (Scheme 13; route C) or Wittig reaction (Scheme 13, route D) [57].

**Scheme 14:**
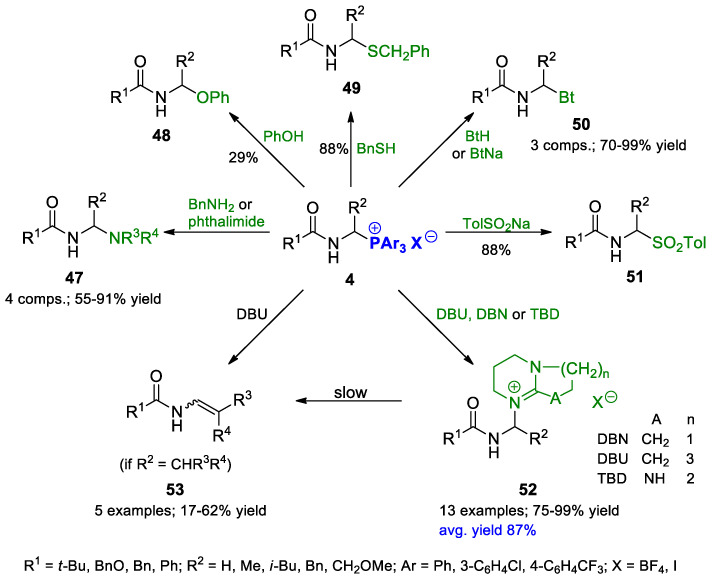
Reactivity of 1-(*N*-acylamino)alkyltriarylphosphonium salts **4** with nucleophiles.

In the years between 2012 and 2021, Mazurkiewicz (triphenylphosphonium derivatives) and then Adamek (phosphonium salts with weakened C_α_-P^+^ bond) explored the possibility of α-amidoalkylation of various other heteronucleophiles (Scheme 14) [46,51,58]. They demonstrated that, under appropriate conditions, *N*-protected 1-aminoalkyltriarylphosphonium salts **4** react with a wide variety of nucleophiles including mercaptans (PhCH_2_SH), phenol (PhOH), amines (PhCH_2_NH_2_), phthalimide, benzotriazole (BtH) or its salts (BtNa), and sodium aryl sulfinates (Ar^2^SO_2_Na) [46,51].

Initially, reactions were carried out at an elevated temperature (60 °C) in the presence of Hünig’s base (for 1-(*N*-acylamino)alkyltriphenylphosphonium salts **4**, Ar = Ph, Scheme 14) [51]. The use of 1-(*N*-acylamino)alkyltriarylphosphonium salts **4** with a weakened C_α_-P^+^ bond strength (Ar = 3-C_6_H_4_Cl, 4-C_6_H_4_CF_3_, Scheme 14) made it possible to conduct these reactions at room temperature without the use of catalysts [46].

The extraordinary α-amidoalkylating properties of 1-(*N*-acylamino)alkylphosphonium salts **4** also allow the α-amidoalkylation of “non-nucleophilic” bases, such as DBU (1,8-diazabicyclo(5.4.0)undec-7-ene), DBN (1,5-diazabicyclo(4.3.0)non-5-ene) or TBD (1,5,7-triazabicyclo(4.4.0)dec-5-ene; Scheme 14). The corresponding 1-(*N*-acylamino)alkylamidinium or guanidinium salts **52** are products in these reactions. They can be isolated but show limited stability; for example, salts **52** with a hydrogen at the β-position underwent transformation to enamides **53**. As shown, enamides **53** can also be obtained directly from phosphonium salts **4** with an appropriate structure (hydrogen at the β-position) by an elimination reaction (Scheme 14) [58].

Interestingly, the 1-(*N*-acylamino)alkylphosphonium salts **4** can be converted to other α-amidoalkylating agents such as *N*-[1-(benzotriazol-1-yl)alkyl]amides **50** or α-amido sulfones **51**. So far, they have been synthesized mainly in a three-component condensation of aldehyde with an amide-type substrate (amides, lactams, urea derivatives, etc.) in the presence of benzotriazole (BtH) or aryl sulfinates, respectively [12,20].

**Scheme 15:**
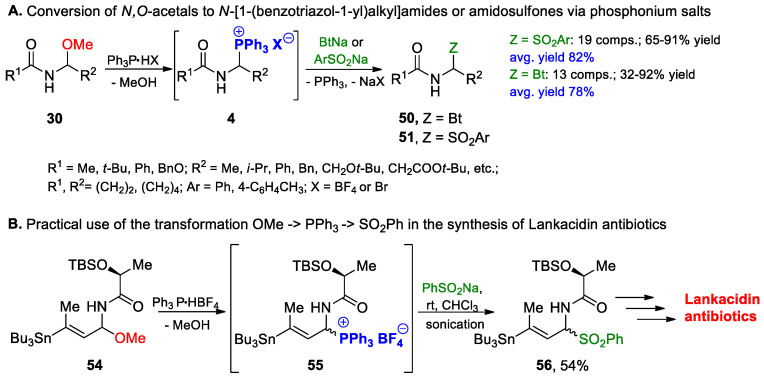
The synthetic use of the transformation of *N*,*O*-acetals to *N*-[1-(benzotriazol-1-yl)alkyl]amides **50** or α-amido sulfones **51, 56** via phosphonium salts.

The proposed methodology extended the base of raw materials with *N,O*-acetals **30** obtained from α-amino acids or amide-type substrates in electrochemical oxidation (alkoxylation). As shown, 1-(*N*-acylamino)alkylphosphonium salts **4** do not have to be isolated in this type of transformation (Scheme 15A, see also Section 2.1.1, Scheme 7) [47,59].

Such transformations gained attention and were used for the preparation of substrates for α-amido sulfone-based intermolecular Mannich addition in the stereodivergent synthesis of lankacyclinol (Lankacidin antibiotics; Scheme 15/B) [60,61,62].

**Scheme 16:**
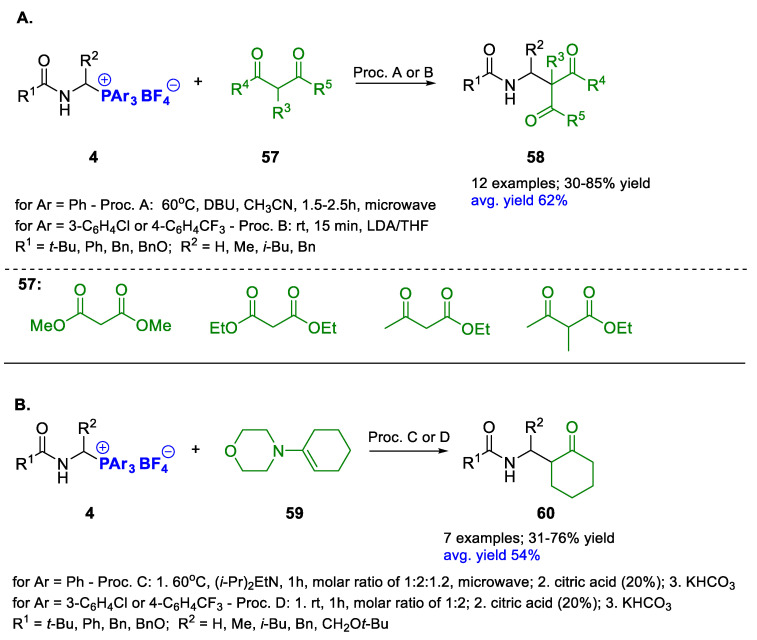
Conditions and yields for reactions of 1-(*N*-acylamino)alkyltriarylphosphonium salts **4** with 1,3-dicarbonyl compounds (**A**) or 1-morpholinocyclohexene (**B**).

High reactivity of 1-(*N*-acylamino)alkylphosphonium salts **4** is also revealed in reactions with *C*-nucleophiles leading to the formation of β-aminocarbonyl systems **58** and **60**. In the case of 1,3-dicarbonyl compounds **57** (dimethyl or diethyl malonate, ethyl acetoacetate, and ethyl 2-methylacetoacetate), it was necessary to use bases (DBU or LDA-lithium diisopropylamide) as catalysts to produce enolate anions [46,51]. However, the use of 1-(*N*-acylamino)alkyltriarylphosphonium salts **4** with a weakened C_α_-P^+^ bond made it possible to conduct the reaction under slightly milder conditions (Scheme 16/A). This was similar in the reaction with 1-morpholinocyclohexene **59**; replacing the triphenylphosphonium residue (Ar = Ph) with a triarylphosphonium group (Ar = 3-C_6_H_4_Cl or 4-C_6_H_4_CF_3_) facilitates the transformation (Scheme 16B) [46,51].

**Scheme 17:**
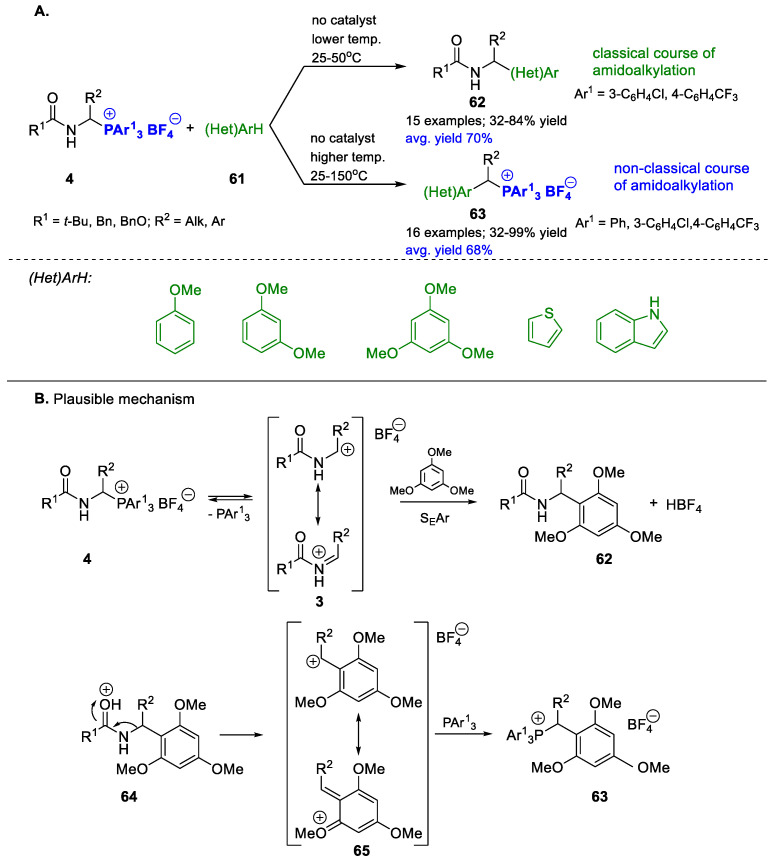
Reaction of 1-(*N*-acylamino)alkyltriarylphosphonium salts **4** with aromatic compounds: (**A**) synthetic routes, (**B**) plausible mechanism.

In 2018, Adamek et al. examined the reactivity of 1-(*N*-acylamino)alkyltriarylphosphonium salts **4** towards various aromatic systems (Scheme 17/A). It was demonstrated that phosphonium salts **4** react with arenes or heteroarenes under non-catalytic conditions. Reactions of triphenylphosphonium salts **4** (Ar = Ph, Scheme 17) required an elevated temperature and led to the formation of 1-arylalkylphosphonium salts **63** (non-classical α-amidoalkylation products). In turn, the use of 1-(*N*-acylamino)alkyltriarylphosphonium salts **4** with a weakened C_α_-P^+^ bond made it possible to carry out the transformations to the expected classical products-*N*-(1-arylalkyl)amides **62** at room temperature. Moreover, it was found that 1-arylalkylphosphonium salts **63** are formed from *N*-(1-arylalkyl)amides **62** in the consecutive-type reaction what is included in the plausible mechanism proposed by the authors (Scheme 17/B) [63].

**Scheme 18:**
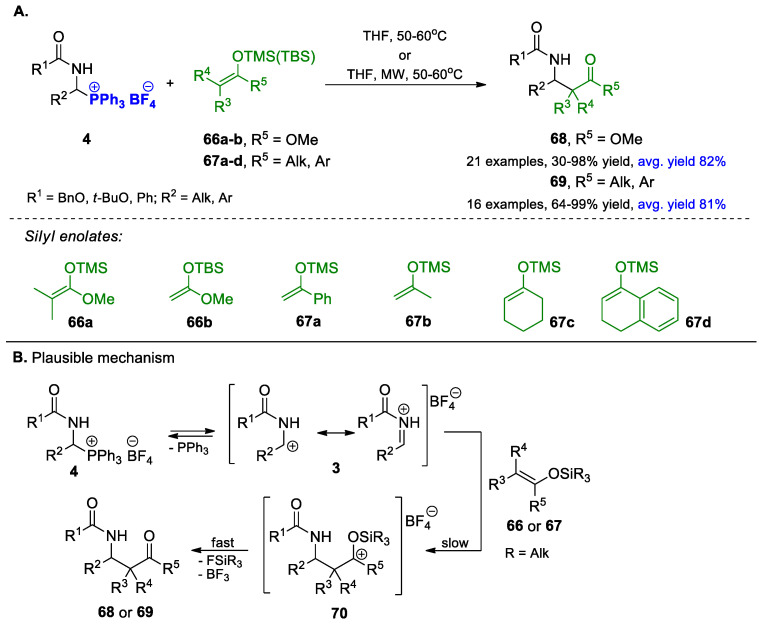
Reaction of 1-(*N*-acylamino)alkyltriarylphosphonium salts **4** with silyl enolates—(**A**) conditions and yields, (**B**) plausible mechanism.

The spontaneous generation of reactive *N*-acyliminium cations from 1-(*N*-acylamino)alkyltriarylphosphonium salts **4** (under catalyst-free conditions) was also used in reactions with silyl enolates **66** or **67**, to provide *N*-protected β-amino esters **68**, as well as *N*-protected β-amino ketones **69** in good to excellent yields (Scheme 18/A). As Październiok-Holewa et al. demonstrated, the process can be carried out in THF at 50 °C or 60 °C using conventional heating or microwave irradiation. The proposed mechanism of the transformation included, in the first stage, the formation of the reactive *N*-acyliminium cation **3**, which further reacts with the silyl enolate to give silyloxy-substituted carbenium ion **70**, which fast undergoes a desilylation reaction to give β-amino carbonyl compounds **68** or **69** (Scheme 18/B) [64].

**Scheme 19:**
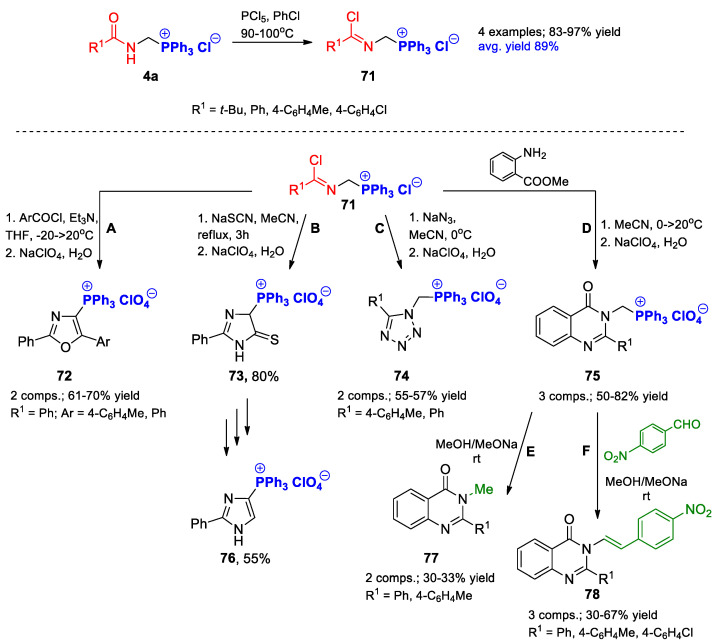
Synthesis of imidoyl chlorides **71** and their further transformations.

1-(*N*-acylamino)alkyltriarylphosphonium salts **4** are bifunctional compounds and their reactivity is not related only to the phosphonium moiety. Already in the 1980s, the transformation of *N*-acylaminomethyltriphenylphosphonium salts **4a** into imidoyl chlorides **71** was described [26]. They turned out to be valuable reagents in cyclization reactions, in which heterocyclic systems such as oxazole, imidazole, tetrazole, or quinazolinone derivatives **72**–**76** can be obtained (Scheme 19/A–D) [26,65,66]. The presence of a triphenylphosphonium group enables further modification of the synthesized heterocycles, which was demonstrated in the example of the quinazolinones **75** (a structural motif of *N*-acylaminoalkylphosphonium salt can also be indicated here). These compounds undergo a reduction under mild conditions (Scheme 19/E). They can also be used as ylide precursors in the Wittig reaction with 4-nitrobenzaldehyde (Scheme 19/F) [26,65,66].

### 2.2. 1-Imidoalkyltriarylphosphonium Salts

**Figure 4:**
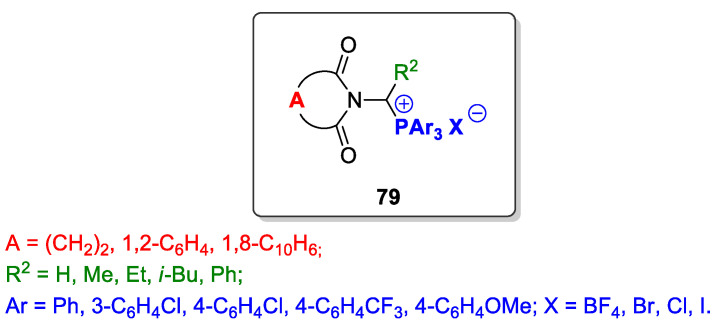
General structure of 1-imidoalkylphosphonium salts **79**.

Structures of the 1-imidoalkylphosphonium salts **79** described in the literature are based on a phthalimide (A = 1,2-C_6_H_4_) or succinimide (A = (CH_2_)_2_) ring (Figure 4). Two electron-withdrawing carbonyl groups connected to the nitrogen atom reduce the electron density at C_α_, thus increasing its electrophilicity. In the α-position there may be hydrogen (R^2^ = H), alkyl (R^2^ = Me, Et, *i*-Bu) or aryl (R^2^ = Ph) substituent. C_α_ is also directly bonded to the triarylphosphonium group PAr_3_ (Ar = Ph, 4-C_6_H_4_Cl, 3-C_6_H_4_Cl, 4-C_6_H_4_CF_3_), which is positively charged and can act as a nucleofugal group.

In most cases, the 1-imidoalkylphosphonium salts are stable solids that can be stored under laboratory conditions for a long time. Interestingly, some of them also show biological activities such as cytotoxic or antimicrobial properties [67,68,69].

#### 2.2.1. Preparation

In general, there is not much information in the literature on the methods for synthesis of 1-imidoalkylphosphonium salts **79**, and most of them concern the simplest ones-imidomethylphosphonium salts (R^2^ = H). To the best of our knowledge, the first attempt to prepare imidomethylphosphonium salts was reported in 1961 by Hellmann and Schumacher [70]. It consisted in the reaction of phthalimidomethyltrimethylammonium iodide with triphenylphosphine in methanol. Such a reaction was later used several times; however, the structure of the substrate and conditions were slightly modified (mainly the solvent, temperature, and reaction time, Table 2).

**Scheme 20:**
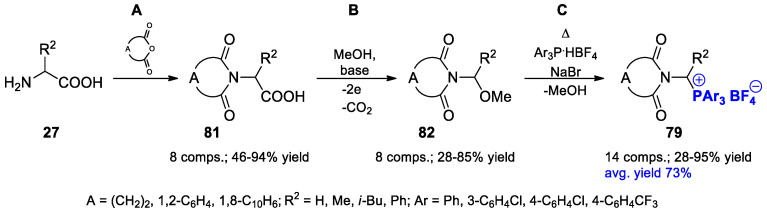
Three-step synthesis of 1-imidoalkylphosphonium salts **79** from amino acids.

After several decades, general methods for the synthesis of imidoalkylphosphonium salts appeared. The first one consisted of three stages: (A) the protection of amino group (from amino acids) by smelting phtalic, succinic or 1,8-naphthalic anhydride with the corresponding amino acid at 140–170 °C; (B) electrochemical decarboxylative α-methoxylation of 1-imidoalkanecarboxylic acids **81**; (C) the displacement of the methoxy group by the triarylphosphine by smelting of the *N*-(1-methoxyalkyl)imides **82** with triarylphosphonium tetrafluoroborates in the presence of NaBr as catalyst (Scheme 20) [73].

**Scheme 21:**
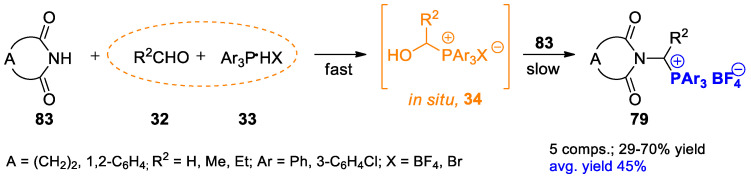
Three-component coupling of aldehydes, imides, and triarylphosphonium salts in the synthesis of 1-imidoalkylphosphonium salts **79**.

Next, 1-imidoalkylphosphonium salts **79** were prepared in the three-component coupling of aldehydes and imides in the presence of triarylphosphonium salts Ar_3_P·HX (Scheme 21). An interesting fact is the formation of an intermediate hydroxyalkylphosphonium salt **34** in situ from aldehyde and triarylphosphonium salt (Ar_3_P·HX) during the reaction (see also Section 2.1.1, Scheme 7) [44].

**Scheme 22:**
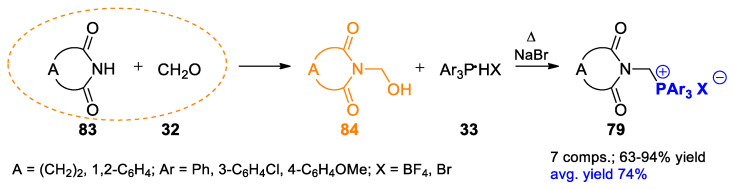
Step-by-step method for the synthesis of 1-imidoalkylphosphonium salts **79** from imides.

As it was demonstrated, 1-imidomethylphosphonium salts **79** can also be obtained in the step-by-step procedure. This time, at first, formaldehyde (reactions with other aldehydes are ineffective) and imides form hydroxymethylimides **84** which, after isolation and purification, are reacted with triarylphosphonium salts **33** (Ar_3_P·HX) in the last stage (Scheme 22). The use of NaBr as a catalyst had a positive effect on the reaction (both on reaction time and yield) when Ar_3_P·HBF_4_ was used (for Ar_3_P·HBr no catalyst is needed) [48].

The last two methods allow for the fast synthesis of 1-imidoalkylphosphonium salts **79** (especially 1-imidomethylphosphonium salts) from readily available substrates, even on a larger scale (5–20 g). Besides, the advantage of both strategies is that they rely on non-electrochemical procedures, thus they are an interesting complement to previously described method.

#### 2.2.2. Synthetic Utilization

**Figure 5:**
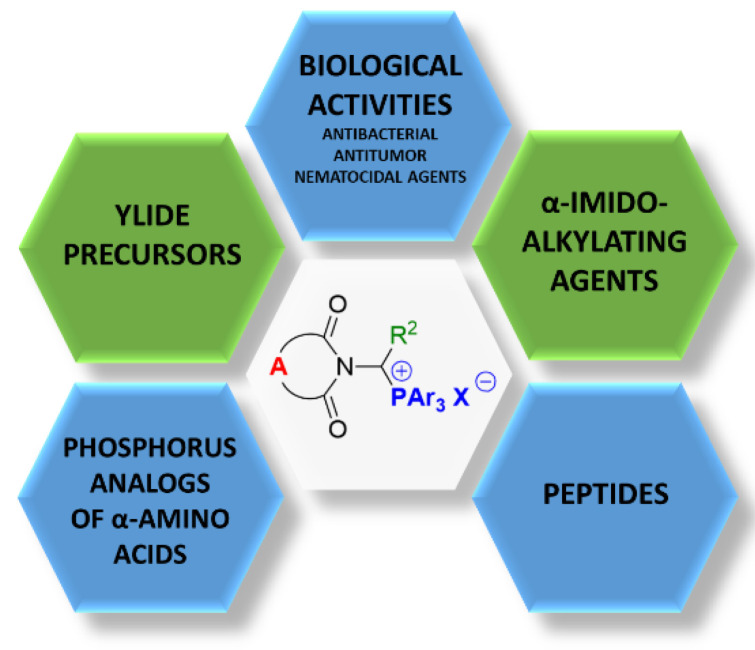
Applications of 1-imidoalkylphosphonium salts **79**.

The most important synthetic applications of 1-imidoalkylphosphonium salts **79** are summarized in Figure 5. Due to certain structural features (dicarbonyl protecting group and thus no NH proton), 1-imidoalkylphosphonium salts **79** can be considered as potential precursors of ylides in the Wittig reaction. These properties of phthalimidomethyltriphenylphosphonium bromide **79** were used by Tan and co-workers in the first stage of multi-step synthesis of compounds **85** and **86**, which are known to modulate the activity of the TAAR_1_ receptor (the trace amine-associated receptor 1, see Table 3) [72].

Recently, the possibilities of using 1-imidoalkylphosphonium salts in imidoalkylation reactions with carbon- or heteronucleophiles have been explored.

**Scheme 23:**
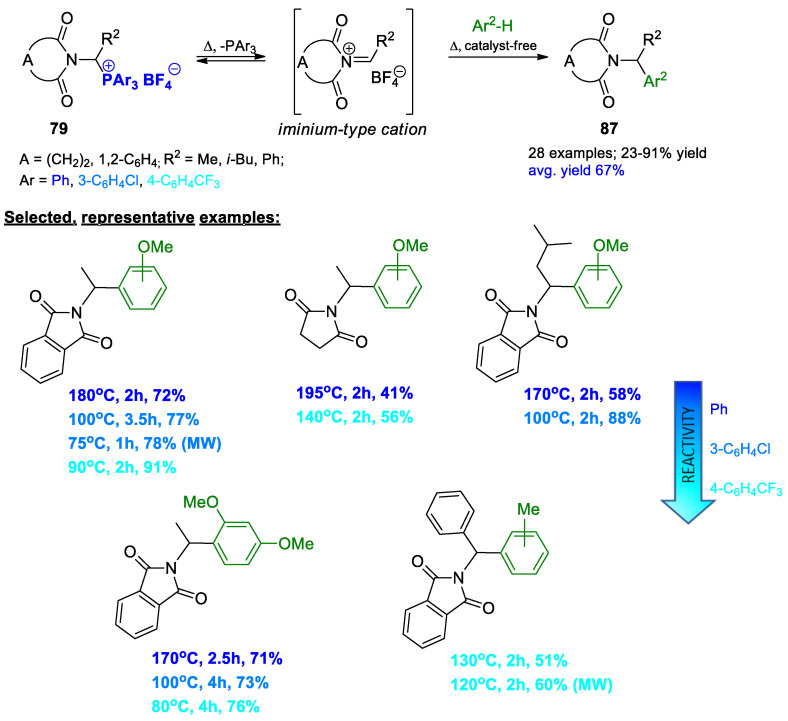
Friedel–Crafts-type reaction of 1-imidoalkylphosphonium salts **79** with various aromatic compounds–conditions and yields (MW–microvawe assisted reaction).

In 2017, the Friedel–Crafts-type reaction of 1-imidoalkylphosphonium salts with various aromatic compounds was described. *N*-(1-arylalkyl)imides **87** were the main products of these transformations (Scheme 23) [73].

**Scheme 24:**
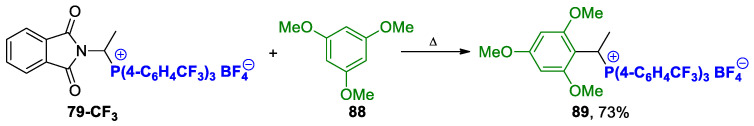
Unusual course of the reaction of 1-imidoalkylphosphonium salt **79**-CF_3_ with a highly activated aromatic system–1,3,5-trimethoxybenzene.

The presence of the dicarbonyl protection increases the electrophilicity of the C_α_. In addition, the use of phosphonium salts which were derivatives of triarylphosphines with electron-withdrawing substituents make it easier to cleave the C_α_-P^+^ bond (first step of the reaction, Scheme 23). Such structural modifications facilitated reactions with aromatic systems, also with weakly activated anisole and toluene (see Scheme 23 and compare the relation between the required reaction temperature and the type of phosphonium moiety). It is worth noting that, contrary to the reaction of the 1-(*N*-acylamino)alkylphosphonium salts **4** with arenes described in this review (Section 2.1.2), no consecutive reaction leading to the so-called non-classical α-amidoalkylation products (1-arylalkylphosphonium salts **63**, see also Scheme 17) was observed. The only exception was the reaction of phosphonium salts **79**-CF_3_ with 1,3,5-trimethoxybenzene (Scheme 24).

1-Imidoalkylphosphonium salts have also been used in the synthesis of imidoalkanephosphonates, imidoalkanephosphinates, and imidoalkylphosphine oxides. Generally, these compounds exhibit interesting biological properties, including antibacterial and antifungal activities or can be used in the synthesis of many bioactive compounds such as phosphapeptides (acting as enzyme inhibitors), oligonucleotides, cytotoxic agents (for example Cryptophycin 52) or 2,4,5-imidazolidinetriones (herbicides and plant growth regulators) [74,75].

**Scheme 25:**
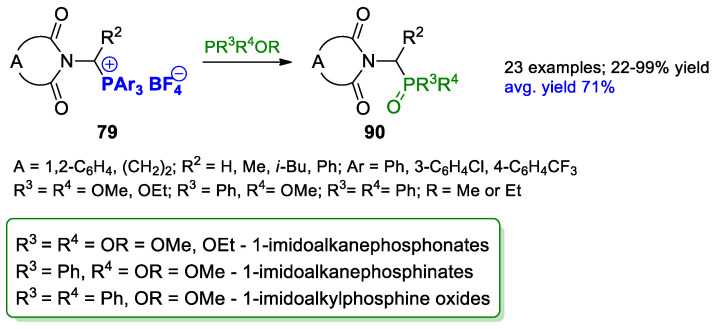
Michaelis–Arbuzov-type reaction of 1-imidoalkylphosphonium salts **79** with selected phosphorus nucleophiles.

The strategy for preparation of P-compounds **90** from phosphonium salts **79** was based on the Michaelis–Arbuzov-type reaction with the appropriate phosphorus nucleophiles (Scheme 25) [76].

It was observed that the reactivity of phosphonium salts **79** strongly depends on their structure. Good yields were obtained only from 1-(*N*-phthalimido)alkylphosphonium salt derivatives of tris(3-chlorophenyl)phosphine and tris(4-trifluoromethylphenyl)phosphine. However, a relatively large excess of phosphorus nucleophile and the addition of methyltriphenylphosphonium iodide (MePPh_3_^+^I^−^) as a catalyst that can facilitate the reaction were required (the most preferred molar ratio of reagents is 1:10:0.25 of phosphonium salt:*P*-nucleophile:catalyst).

### 2.3. N-acyl-1-phosphonio-α-amino Acid Esters

**Figure 6:**
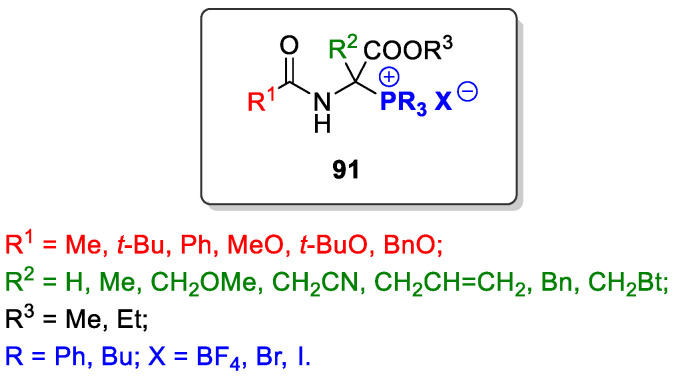
General structure of *N*-acyl-1-phosphonio-α-amino acid esters **91**.

The general structural formula of *N*-acyl-1-phosphonio-α-amino acid esters **91** is shown in Figure 6. In most cases, structures of this kind of phosphonium salts described in the literature are based on a glycinate skeleton (R^2^ = H), although derivatives of other proteinogenic and non-proteinogenic α-amino acids, containing in the α position alkyl (R^2^ = Me, CH_2_OMe, CH_2_CN, CH_2_CH=CH_2_) or alkyl-aryl substituent (R^2^ = CH_2_Ph, CH_2_Bt) are also known. C_α_ is most often directly bonded to the positively charged triphenylphosphonium group (R = Ph), and less often tributhylphosphonium group (R = Bu). In the structure of the phosphonium salts in question, the carboxyl group is protected as an ethyl or methyl ester (R^3^ = Me, Et), while the protected amino group is present as an amide (R^1^ = Me, *t*-Bu, Ph) or carbamate (R^1^ = MeO, *t*-BuO, PhCH_2_O) moiety. The most common counterion to the positively charged phosphonium group is the tetrafluoroborate, bromide or iodide anion (X = BF_4_, Br, I).

#### 2.3.1. Preparation

**Scheme 26:**
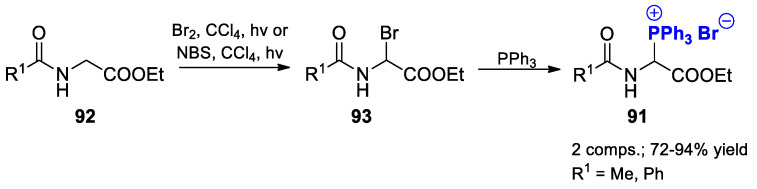
Method for the synthesis of *N*-acyl-1-triphenylphosphonioglycinate bromides **91** from glycine derivatives **92** via 1-bromoglycinates **93**.

For a wide group of compounds belonging to *N*-acyl-1-triphenylphosphonio-α-amino acid esters **91**, *N*-acyl-1-triphenylphosphonioglycinates (R^2^ = H) are the best known ones. They were prepared for the first time in 1983 by Kober and Steglich from ethyl *N*-acyl-1-bromoglycinates **93** by their reaction with triphenylphosphine. The starting 1-bromoglycine derivatives **93** were previously obtained in situ in the reaction of photochemical bromination of *N*-acylglycine ethyl esters **92** with bromine or *N*-bromosuccinimide carried out in tetrachloromethane (Scheme 26) [77].

**Scheme 27:**
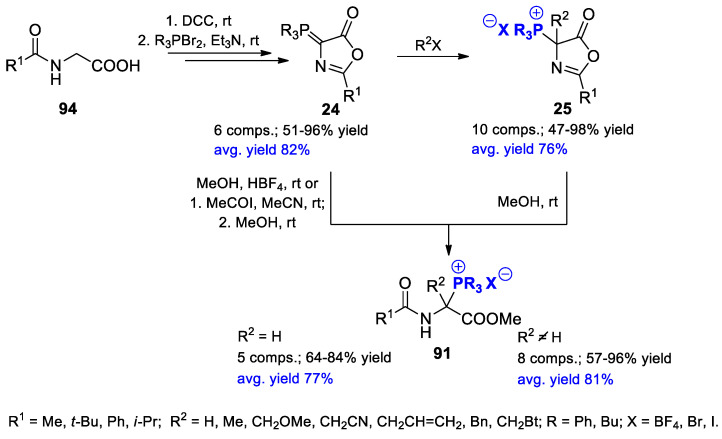
Synthesis of *N*-acyl-1-triphenylphosphonio-α-amino acid esters **91** via phosphoranylidene-5(4*H*)-oxazolones **24**.

In 1996, Mazurkiewicz and Pierwocha developed a simple route for the transformation of *N*-acylated glycine **94** into the 4-phosphoranylidene-5(4*H*)-oxazolones **24** [40]. The corresponding 5(4*H*)-oxazolone, obtained here as an intermediate in the reaction of the starting compound with DCC (*N*,*N*′-dicyclohexylcarbodiimide), is phosphorylated in situ with dibromotriphenylphosphorane (R_3_PBr_2_) in the presence of triethylamine. The resulting phosphoranylidene-5(4*H*)-oxazolones **24** can be further effectively converted into *N*-acyl-1-triphenylphosphonioglycinates (R^2^ = H), as well as esters of other *N*-acyl-1-triphenylphosphonio-α-amino acids **91** (Scheme 27).

However, the most convenient procedure for the synthesis of *N*-acyl-1-triphenylphosphonioglycinate tetrafluoroborates (**91**, X = BF_4_) is to treat a solution of phosphoranylidene-5(4*H*)-oxazolones **24** in methanol with an ethereal solution of tetrafluoroboric acid [78]. An alternative method for the synthesis of *N*-acyl-1-triphenylphosphonioglycinates with an iodide counterion (**91**, X = I) is a two-stage procedure that consists in the reaction of phosphoranylideneoxazolone **24** with acetyl iodide performed in acetonitrile, followed by the subsequent reaction of the acylation product with methanol [78,79]. Similarly, the synthesis of *N*-acyl-1-triphenylphosphonio-α-amino acids **91** with an alkyl substituent at the α-position (R^2^ ≠ H) by alkylation of 4-phosphoranylidene-5(4*H*)-oxazolones **24** with alkyl halides [80], and the next opening of the oxazolone ring under the treatment with methanol or methanol in the presence of an acidic catalyst was also described (Scheme 27) [81].

**Scheme 28:**
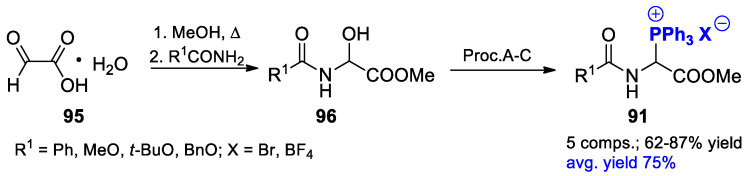
Methodology for the synthesis of *N*-acyl-1-triphenylphosphonioglycinates **91** via α-hydroxyglycinates. Reagents and conditions: Procedure A: Ph_3_P·Br_2_, Et_3_N, Ph_3_P, DCM, rt; Procedure B: DCC, Ph_3_P·HBF_4_, Ph_3_P, DCM, rt; Procedure C: DEAD (diethyl azodicarboxylate), Ph_3_P·HBF_4_, Ph_3_P, THF, rt.

In 2004, three methods for the tranformation of *N*-alkoxycarbonyl-1-hydroxyglycinates **96** into especially interesting *N*-alkoxycarbonyl-1-triphenylphosphonioglycinates **91** (R^1^ = MeO, *t*-BuO, BnO) were developed by Mazurkiewicz et al. The proposed synthetic routes included the following transformations: phosphorylation of *N*-alkoxycarbonyl-1-hydroxyglycinates **96** with Ph_3_P·Br_2_ in the presence of Et_3_N (Procedure A), the reaction of *N*-alkoxycarbonyl-1-hydroxyglycinates **96** with DCC and Ph_3_P·HBF_4_ in the presence of Ph_3_P as a catalyst (Procedure B), and a new kind of the Mitsunobu reaction using Ph_3_P·HBF_4_ as a nucleophile conjugated acid (Procedure C, Scheme 28) [82].

#### 2.3.2. Synthetic Utilization

**Figure 7:**
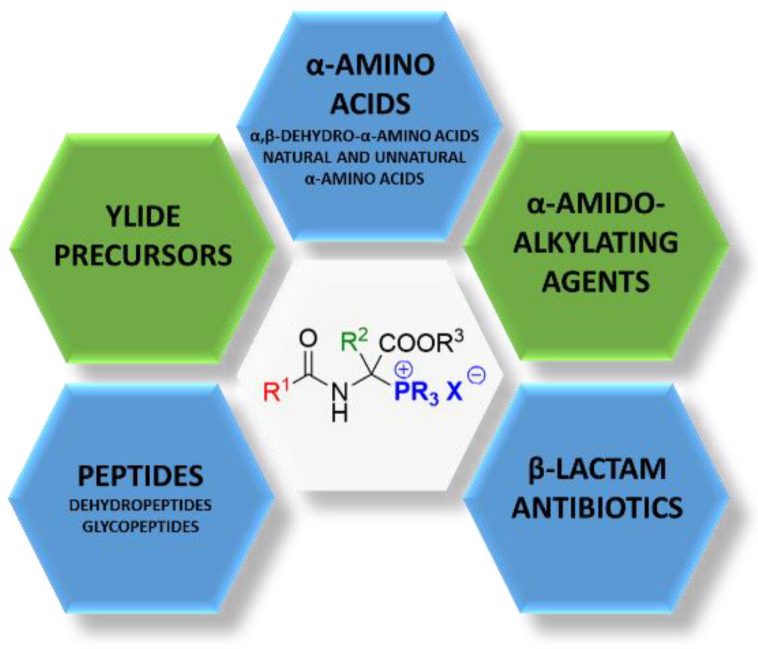
Applications of *N*-acyl-1-phosphonio-α-amino acid esters **91**.

*N*-Acyl-1-triphenylphosphonio-α-amino acid esters **91** are, in most cases, crystalline compounds, stable at room temperature, moderately sensitive to moisture, and well soluble in DCM and MeCN, but insoluble in diethyl ether. They can be easily purified by crystallization consisting of dissolution in DCM or MeCN at room temperature and precipitation with diethyl ether [78,79,80,81,82]. It is worth emphasizing that they are easily accessible from *N*-acylglycine even at kilogram scale (Scheme 26 and Scheme 27). All of these features of *N*-acyl-1-triphenylphosphonio-α-amino acid esters, as well as their diverse reactivity make these compounds interesting reagents in organic syntheses (Figure 7).

**Scheme 29:**
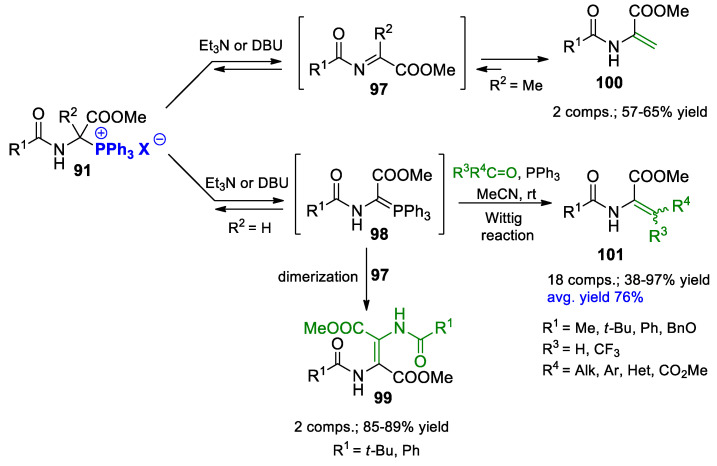
Various pathways for synthetic applications of *N*-acyl-1-triphenylphosphonio-α-amino acid esters **91** in the presence of a base (Et_3_N or DBU).

The directions of *N*-acyl-1-triphenylphosphonio-α-amino acid esters reactivity, and thus, the possibility of their further applications, were recognized during comprehensive research on their behavior in the presence of organic bases [83]. Reactions of *N*-acyl-1- triphenylphosphonio-α-amino acid methyl esters **91** with DBU and triethylamine were investigated then as the crucial step of the base catalysed displacement of the triphenylphosphonium group by various nucleophiles. Initially, this was observed by Kober, and Steglich, and later confirmed by Mazurkiewicz and Grymel, that *N*-acyl-1-triphenylphosphonioglycinates **91**, upon treatment with bases, were converted into a mixture of the corresponding *N*-acyliminoacetate **97** and *N*-acyl-1-triphenylphosphoranylideneglycinate **98**. Both the iminoacetate **97** and the ylide **98** turned out to be highly reactive, instable compounds that remained in an equilibrium and reacted slowly with each other providing the fumaric acid derivative **99**. In the case of *N*-acyl-1-triphenylphosphonio-α-amino acid esters **91** with the quaternary α-carbon, the α-substituted homologues of *N*-acyliminoacetate **97** can undergo further tautomerization into the corresponding enamine **100** (Scheme 29) [83].

The application of *N*-acyl-1-triphenylphosphonioglycinates **91** as the precursors of phosphonium ylides **98** in the Wittig reaction with aliphatic or aromatic aldehydes in the presence of Et_3_N allowed the development of a simple and efficient procedure for the synthesis of α,β-dehydro-α-amino acid derivatives **101** (Scheme 29) [84].

**Scheme 30:**
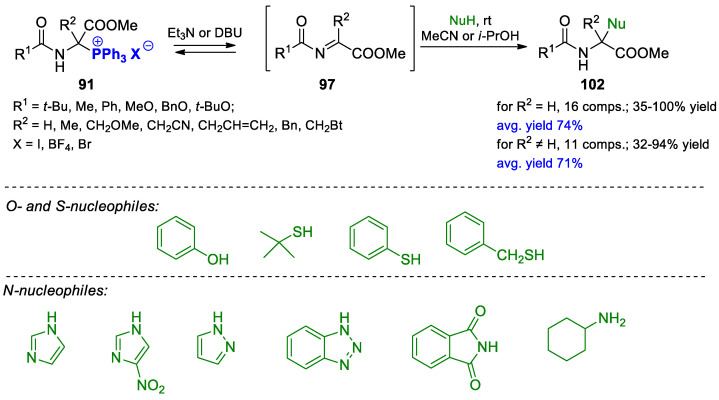
Synthetic applications of *N*-acyl-1-triphenylphosphonio-α-amino acid esters **91** in reactions with heteronucleophiles.

On the other hand, methods for the displacement of the triphenylphosphonium group with a variety oxygen, sulfur, nitrogen and carbon nucleophilic agents, consisting in the addition of a nucleophile to the activated C=N double bond of the *N*-acylimino intermediate **97**, opened up new routes for the synthesis of biologically important natural and unnatural non-proteinogenic α-amino acids by double functionalization of the glycine α-position with electrophilic and nucleophilic reagent (Scheme 30) [78,79,81].

**Scheme 31:**
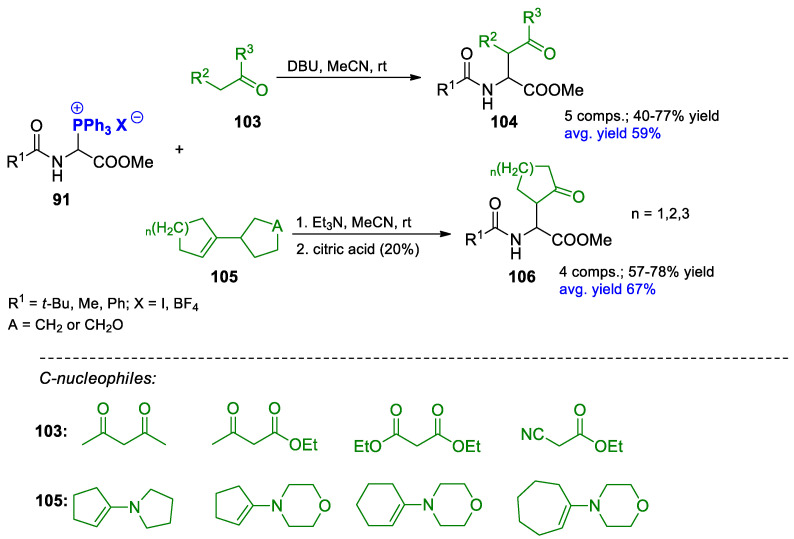
Synthetic applications of *N*-acyl-1-triphenylphosphonio-α-amino acid esters **91** in reactions with *C*-nucleophiles.

*N*-acyl-1-triphenylphosphonio-α-amino acid esters **91** react easily with a wide variety of oxygen, sulphur and nitrogen nucleophiles including phenol (PhOH), mercaptans (*t*-BuSH, PhSH, PhCH_2_SH), imidazole, 4-nitroimidazole, pyrazole, benzotriazole, phthalimide, cyclohexylamine (Scheme 30) [78,81] and two kinds of carbon nucleophiles: enolates **103** of activated carbonyl compounds or enamines **105** (Scheme 31) [79]. Reactions were conducted in acetonitrile or methanol at room temperature in the presence of DBU or triethylamine, and the corresponding α-amino acid derivatives **102**, **104**, and **106** (including α,α-difunctionalized derivatives) were usually obtained in good to excellent yields [78,79,81].

This great interest in natural non-proteinogenic α-amino acids results from their diverse biological activities as antibiotics, pharmaceuticals, natural pesticides, and growth regulators, as well as their use in the synthesis of enzymes, hormones, new chemotherapeutics, synthetic immunostimulants, and other protein structured compounds [85,86]. The importance of α,α-disubstituted α-amino acids has been comprehensively discussed by many authors [87,88].

**Scheme 32:**
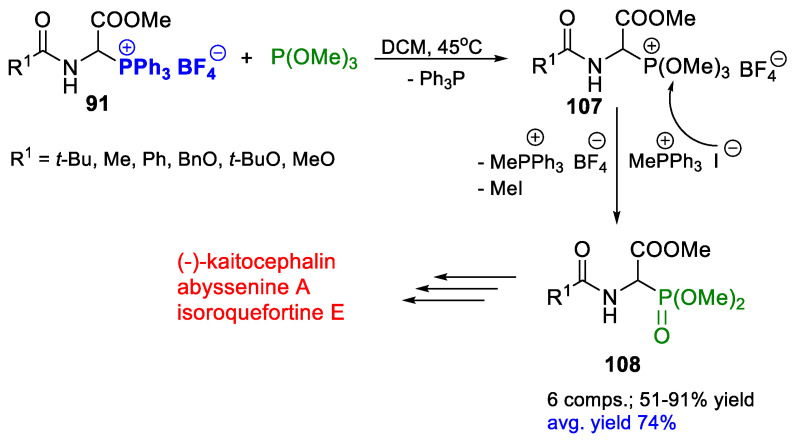
Transformation of *N*-acyl-1-triphenylphosphonioglycinate tetrafluoroborates **91** into *N*-acyl-α-(dimethoxyphosphoryl)glycinates **108** and selected examples of their further use in the synthesis of biologically active compounds.

As demonstrated by Mazurkiewicz and Kuźnik, *N*-acyl-1-triphenylphosphonioglycinate tetrafluoroborates **91** are also convenient starting compounds for the transformation into *N*-acyl-α-(dialkoxyphosphoryl)glycinates **108** by the Michaelis–Arbuzow-type reaction with trimethylphosphite in the presence of methyltriphenylphosphonium iodide as a catalyst (Scheme 32) [89]. Among others, α-(dialkoxyphosphoryl)glycinates became the crucial synthetic tool commonly used for the synthesis of many natural products (including β-lactam antibiotics) or α,β-dehydro-α-amino acids by the Wadsworth-Emmons reaction [90,91,92,93,94,95,96]. As is known, hydrogenation of the latter compounds using chiral catalysts is considered to be one of the most general methods for the enantioselective synthesis of α-amino acids, including non-proteinogenic α-amino acids of diverse biological activities [97,98,99,100].

**Scheme 33:**
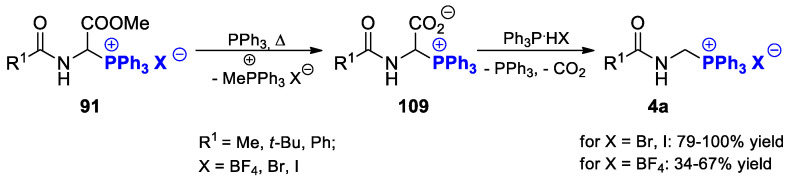
Thermal stability of N-acyl-1-triphenylphosphonio-α-amino acid esters **91**.

Although *N*-acyl-1-triphenylphosphonioglycinates **91** are relatively stable, they undergo interesting transformations at high temperatures. Thermogravimetric investigations revealed that during the process of the melting of salts **91**, they underwent demethoxycarbonylation, providing *N*-acylaminomethyltriphenylphosphonium salts **4a** (18–50%), along with methyltriphenylphosphonium salts (22–68%). When this reaction was performed in the presence of Ph_3_P and Ph_3_P∙HX (X = Br, BF_4_, I) the process of demethoxycarbonylation for *N*-acyl-1-triphenylphosphonioglycinate bromides and iodides (X = Br, I) occured at 95–130 °C in good to excellent yields (79–100%); whereas for *N*-acyl-1-triphenylphosphonioglycinate tetrafluoroborates **91** (X = BF_4_) as starting compounds, the analogous transformation occured at about 170–175 °C, giving the corresponding phosphonium tetrafluoroborates **4a** in much lower yields (34–67%; Scheme 33) [101]. The practical significance of this process is due to the fact that the obtained 1-(*N*-acylamino)alkyltriphenylphosphonium salts **4a** can be used as valuable α-amidoalkylating agents (see also Section 2.1.2).

**Scheme 34:**
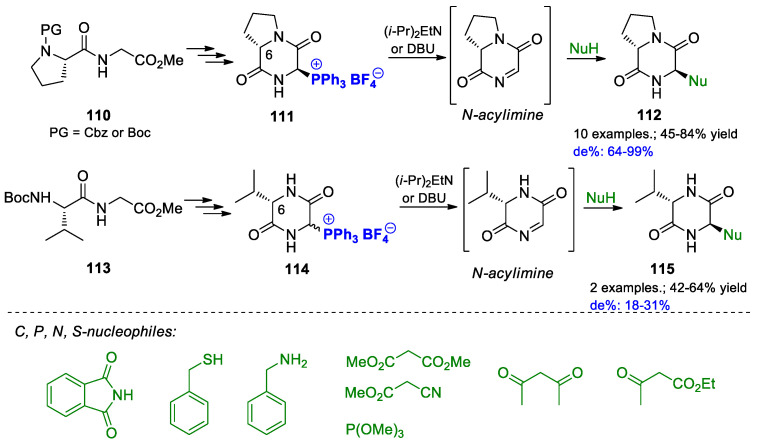
Synthetic use of *3-triphenylphosphonio-2,5-piperazinedione* **111***,* **114***-chiral glycine cation equivalents*.

The crucial structural motif for *N*-acyl-1-triphenylphosphonio-α-amino acid esters (amino, phosphonium and carbonyl groups bonded to the same carbon atom) can be a part of more complex systems. In this regard, 3-triphenylphosphonio-2,5-piperazinedione **111**, **114** can be considered as structurally similar compounds to the phosphonium salts **91**. They can be obtained from dipeptides in multistep procedure described by Mazurkiewicz and Gorewoda in 2011 [102]. The retention of configuration (position 6) results in the formation of chiral glycine cation equivalents **111**, **114** which can be used for a diastereoselective nucleophilic substitution of the triphenylphosphonium group with *S*-, *N*-, *P*-, and *C*-nucleophiles (Scheme 34). Reactions were conducted at 0 or 25 °C in the presence of a base (*i*-Pr_2_EtN or DBU) and were particularly effective (high yields and high de%) for the proline derivative **111** [102].

## 3. Conclusions

1-Aminoalkylphosphonium derivatives are, in most cases, crystalline compounds, stable at room temperature and well soluble in chloroform, dichloromethane or acetonitrile, which makes them easy to store (even for a long time) and convenient to use reagents. On the other hand, they show remarkable reactivity especially towards various kinds of nucleophiles (both carbon- and heteronucleophiles). Moreover, the structure of such phosphonium salts is easy to modify by changing the *N*-protecting group or introducing electron-withdrawing or electron-donating substituents to the phosphonium moiety by using appropriately modified phosphines in the key stage of the synthesis. It allows for the control and, more interestingly, the targeting of the reactivity of these phosphonium compounds (α-amidoalkylation reaction vs. Wittig reaction).

**Figure 8:**
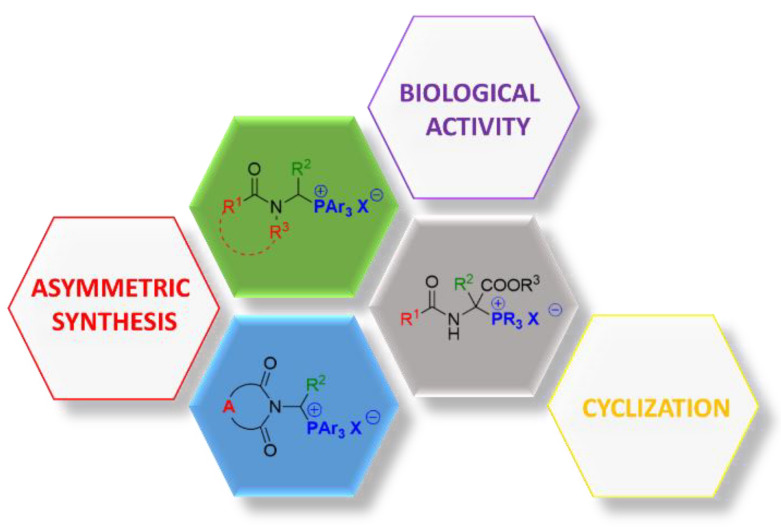
1-Aminoalkylphosphonium derivatives—new challenges.

All these factors make the 1-aminoalkylphosphonium derivatives an interesting group of “smart-reagents” with great potential as precursors of reactive intermediates such as *N*-acyliminium-type cations (generated without the need for any catalysts), or ylides. This was used in the synthesis of such compounds as phosphorus analogs of α-amino acids, β-aminocarbonyl systems, 1-arylalkylphosphonium salts or α,β-dehydro-α-amino acids, which are very important because of their valuable biological and chemical properties. However, most of the described reactions were intermolecular (did not lead to cyclization) and were not conducted in a stereocontrolled manner. These two aspects require further research because such transformations are of great importance in the synthesis of natural, biologically active compounds. It seems that, especially in this field, the easy ability to control the C_α_-P^+^ bond strength and introduce structural modifications within phosphonium salts may be crucial (Figure 8—new challenges/asymmetric synthesis/cyclization). Studies on cyclization and stereocontrol of reactions involving 1-aminoalkylphosphonium salts are in progress.

It is worth adding that, not only many of the described compounds obtained from 1-aminoalkylphosphonium salts derivatives, but also some phosphonium salts themselves show interesting biological properties. However, in this case, the area of potential application should also be much more explored. 1-Aminoalkylphosphonium salts derivatives can be an ideal tool for the modification of already known structures with proven biological activity. Furthermore, recent reports on mitochondria-targeted phosphonium salts inspire the design and synthesis of molecular hybrids or conjugates that will use the targeting properties of the triphenylphosphonium (TPP) group, its biological properties, or both (Figure 8—new challenges/biological activity).

We hope that the presented data will encourage further research on 1-aminoalkylphosphonium salt derivatives and will contribute to discovering their full potential.

## Figures and Tables

**Table 1:** Summary of characteristics for the most important precursors of α-amidoalkylating agents **1**.

Structure of Precursor	Summary of Characteristics	Examples of Use in α-Amidoalkylation (Selected Research or Review Literature) ^a^
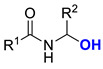	limited structural diversity, limited reactivity, parent compounds for the other α-amidoalkylating agents, activation with acidic catalysts, synthesis from amides (or imides) and aldehydes (mostly in situ)—only *N*-hydoxymethylamides (or -imides) can be easily isolated	[3,4,6,7,8,9,10,11,12]
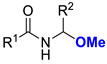	limited reactivity, high structural diversity, activation with acidic catalysts, main synthesis methods based on electrochemical alkoxylation	[5,6,7,8,9,12,13,14]
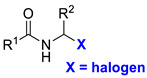	high reactivity, rather low yields in α-amidoalkylation reactions (lots of by-products), difficulties in the preparation, purification and storage	[6,7,8,9,12]
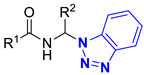	high reactivity (good leaving group), high structural diversity, activation with acidic catalysts, easy to use and storage, diverse methods of synthesis, broad scope of application	[8,9,12,16,17,18,19]
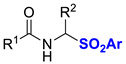	high reactivity (good leaving group), high structural diversity, activation with acidic catalysts, easy to use and storage, diverse methods of synthesis, broad scope of application, currently the most popular and convenient	[8,9,12,20,21,22,23]

^a^ Selected examples aimed at showing the most recent interest in α-amidoalkylation reactions.

**Table 2:**
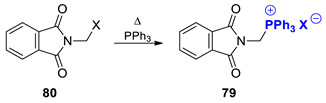
Conditions and yields for the synthesis of phthalimidomethylphosphonium salts **79**.

Entry	Substrate 80	Solvent	Conditions	Yield of 79, %	Refs.
	**X**				
1	(Me_3_N)^+^ I^-^	methanol	reflux, 4 h	58	[70]
2	Cl	benzene	reflux, 2 h	-	[70]
3	Br	acetone	reflux, 3 min	80	[24]
4	Br	benzene	reflux, 22 h	68	[67,71] ^a^
5	Br	toluene	reflux, 24 h	-	[72]

^a^ Compound 79 (X = Br) is also formed as a by-product in the reaction with Pd(PPh_3_)_4_ (rt, benzene).

**Table 3:**
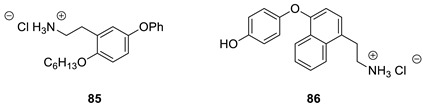
Application of phthalimidomethyltriphenylphosphonium bromide as ylide precursors.

Phosphonium Salt 79	Carbonyl Components	Conditions	Intermediate Compound	Targeted Compound
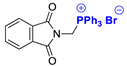	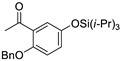	KHMDS, THF, 0 °C→RT, 86%	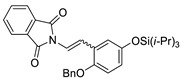	**85**
	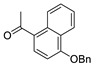	KHMDS, THF, 0 °C→RT, 77%	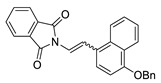	**86**

## Data Availability

Not applicable.
